# Outcomes of surgery for different types of chronic pulmonary aspergillosis: results from a single-center, retrospective cohort study

**DOI:** 10.1186/s12890-022-01836-z

**Published:** 2022-01-19

**Authors:** ChangMing Shen, GaoFeng Qiao, Cheng Wang, Feng Jin, YunZeng Zhang

**Affiliations:** 1 Department of Thoracic Surgery, Shandong Public Health Clinical Center, No.2999, GangXing Xi Road, Jinan, Shandong China; 2 Department of Thoracic Surgery, Shandong Provincial Chest Hospital, Jinan, Shandong China

**Keywords:** Chronic pulmonary aspergillosis, Surgery, Outcome

## Abstract

**Background:**

The reported experience of surgical treatment for chronic pulmonary aspergillosis (CPA) mainly focused on simple aspergilloma (SA), few about other types of CPA. The present study aims to evaluate the outcomes of surgical treatment for different types of CPA.

**Methods:**

We performed a retrospective analysis of 85 patients with CPA who underwent surgery from 2014 to 2020 at Shandong Provincial Chest Hospital. The patients were divided into four types, including SA, chronic cavitary pulmonary aspergillosis (CCPA), chronic fibrosing pulmonary aspergillosis (CFPA), aspergillus nodule (AN). We collected and analyzed the preoperative, perioperative, and postoperative data to evaluate the outcomes of surgical treatment of different types of CPA.

**Results:**

The four groups had similar age (p = 0.22), symptoms (p = 0.36), lesion location (p = 0.09), VATS rate (p = 0.08), recurrence rate (p = 0.95), and had significant difference in surgical procedures (p < 0.01), time of surgery (p < 0.01), intraoperative blood loss (p < 0.01), postoperative complication (p = 0.01). CFPA (P = 0.01), longer surgical time (P = 0.001), and more intraoperative blood loss (P = 0.004) were risk factors of postoperative complication, more intraoperative blood loss (> 400 ml) was the independent risk factor (OR 13.5, 95% CI 1.6–112.1, P = 0.02). 6 patients relapsed after surgery with a recurrence rate of 7.1%. The mean time to relapse was 14.8 months (2–30 months) after surgery. Relapse occurred in 2 SA patients, 3 CCPA, and 1 CFPA, respectively, while none of the AN patients relapsed. No risk factor for recurrence was found.

**Conclusions:**

Surgical resection seems safe and effective in the treatment of SA, AN, CCPA with a low complication and recurrence rate, while surgery for CFPA should be limited to selected patients because of its higher complication rate.

**Supplementary Information:**

The online version contains supplementary material available at 10.1186/s12890-022-01836-z.

## Background

Chronic pulmonary aspergillosis is a pulmonary infection caused by caused by Aspergillus species [1]. Recent reports have shown an increased incidence of CPA in recent years [2–4]. The European Society for Clinical Microbiology and Infectious Diseases (ESCMID) and the European Respiratory Society (ERS) reached an agreement on the rationale and clinical guidelines for diagnosis and management for CPA in 2015 [5]. CPA is categorised as single (simple) pulmonary aspergilloma (SA), chronic cavitary pulmonary aspergillosis (CCPA), chronic fibrosing pulmonary aspergillosis (CFPA), aspergillus nodule (AN), subacute invasive aspergillosis (SAIA).

SA with symptoms is recommended for surgical treatment, while close follow-up with observation is recommended for asymptomatic SA. Treatment for AN differs from clinical symptoms and relevant examinations. Other types of CPA are all first treated by antifungal therapy usually [5, 6]. However, some patients are not sensitive to antifungal therapy and need to use drugs for a long time. Side effects, drug resistance, inevitable recurrence after drug withdrawal, and other problems also come along with them. Surgery offers a chance of cure, but this is limited to selected patients with localized lesions. The reported experience mainly focused on simple aspergilloma, few about other types of CPA.

We believe that surgery is beneficial for certain CPA patients, no matter what type of CPA. What’s more, we have performed surgery for different types of CPA in recent years. Herein, we reviewed our experience with the surgical management of 85 CPA patients in the present study, to evaluate the surgical outcomes of different types of CPA.

## Methods

From 2014 to 2020, 85 patients with CPA underwent surgery at Shandong Provincial Chest Hospital in China. The clinical data were collected retrospectively, including gender, age, comorbidities, symptoms, location and CPA classification, preoperative bronchial artery embolization, preoperative and postoperative antifungal therapy, surgical procedures, intraoperative blood loss, and postoperative complications.

The diagnosis of CPA was based on the criteria of ERS and ESCMID: one or more cavities with or without a fungal ball or nodules present on thoracic imaging for ≥ 3 months, direct evidence of Aspergillus infection (sputum culture), or immunological response to *Aspergillus *spp. (GM (galactomannan) assay, Megazyme Co.), and exclusion of alternative diagnoses. Once diagnosed, voriconazole was administered for at least 2 weeks, except for drug-intolerance or fatal massive hemoptysis.

The surgical indication of CPA was that the main lesions were confined to the ipsilateral lung with the following conditions: massive hemoptysis; persistent or recurrent small amount of hemoptysis; antifungal treatment was ineffective or intolerable; suspected to be lung cancer.

**Fig. 1:**
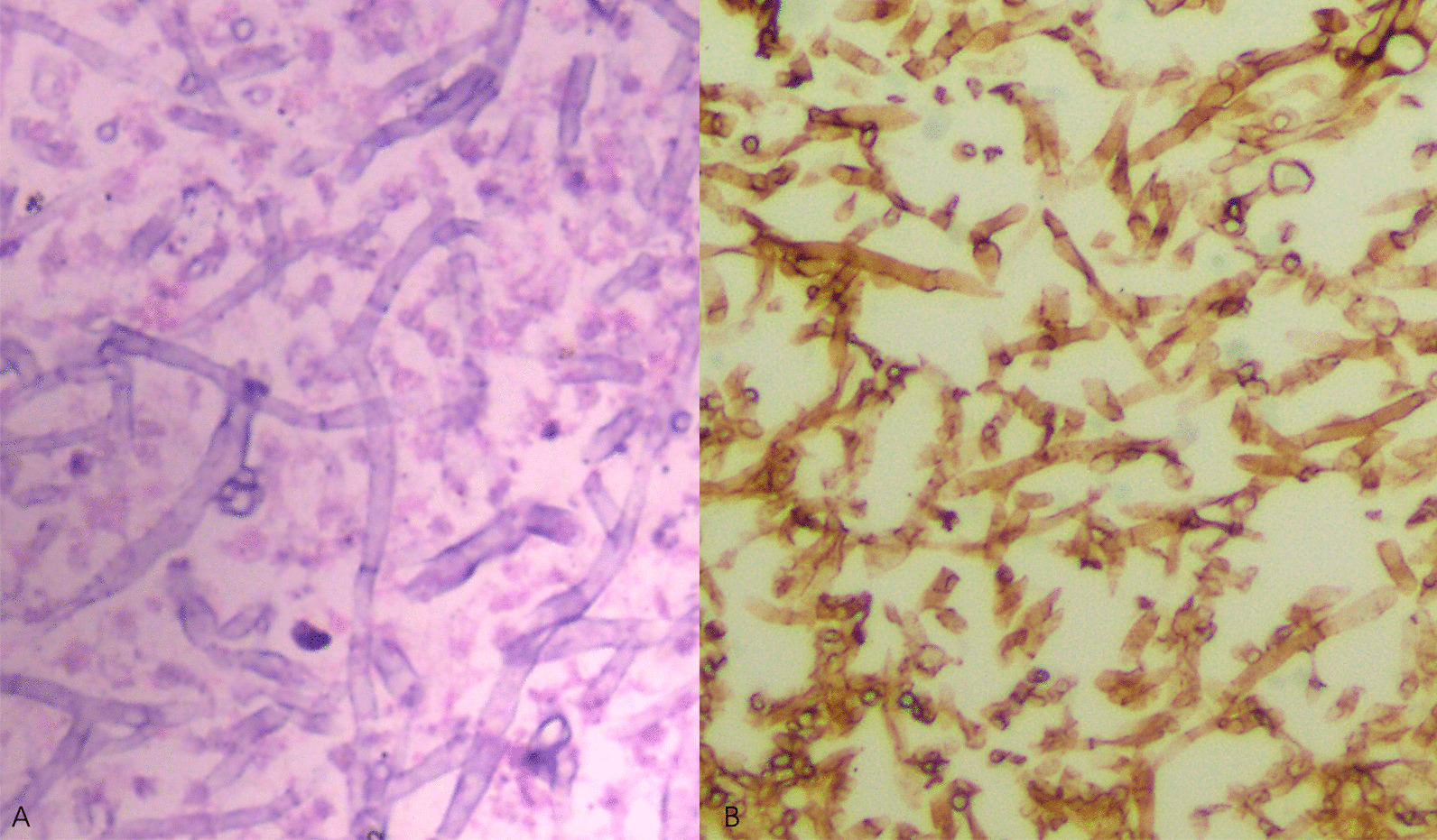
Postoperative pathological images of CPA. **a** Hematoxylin–Eosin staining. **b** Gomori's Methenamine Silver staining

The CPA diagnosis in these patients was pathologically confirmed after surgery (Fig. 1). Voriconazole was administered for at least 2 weeks for SA and AN patients, 3 months for CCPA, and 6 months for CFPA, except for drug-intolerance.

Being a retrospective study, ethics review and patients’ informed consent were waived.

Statistical analyses and data management were performed using SPSS 21.0. Continuous variable data was expressed as the mean ± SD, whereas categorical data was presented as number or percentage. The Pearson chi-square test or continuous correction Chi-square test or Fisher's precision probability test was used to compare categorical variables. Binary logistic regression analysis was used to assess risk factors for postoperative complication and recurrence. Comparisons were considered to be statistically significant when P < 0.05.

## Results

85 patients were enrolled in this study with a mean age of 46.6 years (range 7–74 years), including 50 men (58.8%) and 35 women (41.2%). The general clinical characteristics of the four types of CPA are shown in Table 1. CCPA was the most common type, accounting for 41.2% of CPA, while the percentage of SA was 29.4%, CFPA 15.3%, AN 14.1%. The most common underlying pulmonary disease was pulmonary tuberculosis (n = 44, 51.8%), followed by pulmonary cyst (n = 20, 23.5%). The less common diseases included bronchiectasis (n = 6, 7.1%), tuberculous empyema (n = 3, 3.5%), lung cancer (n = 2, 2.4%), pulmonary bullae (n = 1, 1.2%), and lung abscess (n = 1, 1.2%). 8 patients (9.4%) showed no respiratory disease. The main symptoms included cough and sputum, hemoptysis, which are shown in Table 1.

**Table 1:** Baseline clinical characteristics of CPA

Type of CPA	SA (n = 25)	CCPA (n = 35)	CFPA (n = 13)	AN (n = 12)	P value
*Gender*					0.186
Male	12	23	10	5	
Female	13	12	3	7	
Age (years)	43.64 ± 13.68	45.14 ± 12.97	51.31 ± 18.35	51.75 ± 12.93	0.22
*Symptom*					0.36
Cough and sputum	8	7	5	3	
Hemoptysis	17	25	8	7	
Asymptomatic	0	3	0	2	
Preoperative bronchial artery embolization	1(4%)	5(14.3%)	2(15.4%)	1(8.3%)	0.60
*Main lesion location*					0.09
Right upper lobe	14	15	6	6	
Right lower lobe	0	4	1	3	
Left upper lobe	6	7	0	2	
Left lower lobe	4	5	1	1	
Multiple lobes	1	4	5	0	
*Underlying pulmonary diseases*					< 0.01
Pulmonary tuberculosis	9	24	8	3	
Pulmonary cyst	10	7	0	3	
Bronchiectasis	0	2	2	2	
Tuberculous empyema	0	1	2	0	
Lung cancer	2	0	0	0	
Pulmonary bullae	0	0	1	0	
Lung abscess	0	1	0	0	
No definite underlying diseases	4	0	0	4	
*Fungal cultures*					0.43
Positive	1	3	2	0	
Negative	24	32	11	12	
*GM assay*					
Positive	3	6	4	1	0.41
Negative	22	29	9	11	

The main indications for surgery included hemoptysis (n = 56, 65.9%), failure or intolerance of antifungal therapy (n = 22, 25.9%), suspicion of lung cancer (n = 7, 8.2%). Of the 56 patients with persistent hemoptysis, 2 received emergency surgery because of massive hemoptysis, and 11 suffered from persistent small amount of hemoptysis. And recurrent hemoptysis appeared in 43 patients, of whom 9 accepted bronchial artery embolization to control hemorrhage. However, hemoptysis occurred again in a short time. The mean duration of hemoptysis was 17.3 months (range 4 days–204 months). 7 patients were suspected to have lung cancer, whereas only 2 were confirmed to have lung cancer combined with aspergillosis (1 squamous cell carcinoma, 1 neuroendocrine carcinoma), another 4 were AN, 1 was SA. CPA lesions were most often found in the right upper lobe, and no lesions were found in the right middle lobe.

The surgical procedures are listed in Table 2. More than half of the 85 patients accepted lobectomy (n = 43, 50.6%), and wedge resection (n = 14, 16.5%) was the second most frequent procedure, followed by segmentectomy (n = 13, 15.3%). 4 patients received pleural strip with lobectomy or segmentectomy because of empyema or pleural thickening, and the pneumonectomy was performed for 1 SA patient and 1 CFPA patient. Video-assisted thoracoscopy (VATS) was performed in 37 patients (43.5%).

**Table 2:** Surgical outcomes of CPA

Type of CPA	SA (n = 25)	CCPA (n = 35)	CFPA (n = 13)	AN (n = 12)	P value
*Surgical procedures*					< 0.01
Lobectomy	12	20	6	6	
Segmentectomy	2	7	0	4	
Wedge resection	10	2	0	2	
Lobectomy and Segmentectomy/ *Wedge resection*	0	5	6	0	
Pneumonectomy	1	0	1	0	
Time of surgery (minutes)	161.8 ± 56.3 (95% CI 138.6–185.0)	231.2 ± 97.4 (95% CI 197.7–264.6)	331.5 ± 81.3 (95% CI 282.4–380.6)	264.6 ± 196.9 (95% CI 139.5–389.7)	< 0.01
Intraoperative blood loss (ml)	211.2 ± 222.1 (95% CI 119.5–302.9)	548.6 ± 701.3 (95% CI 307.7–789.5)	1076.9 ± 708.5 (95% CI 648.8–1505.0)	312.9 ± 261.4 (95% CI 146.8–479.0)	< 0.01
VATS	15(60%)	11(31.4%)	4(30.8%)	7(58.3%)	0.08
Postoperative complication	1(4.0%)	2(5.7%)	5(38.5%)	2(16.7%)	0.01
Postoperative antifungal therapy	20 (80%)	24 (68.6%)	11 (84.6%)	11 (91.7%)	0.33
Recurrence	2(8.0%)	3(8.6%)	1(7.7%)	0	0.94

The amount of intraoperative blood loss and time of surgery are shown in Table 1. The SA patients consumed the shortest operation time among the four group’s patients (P = 0.01, P < 0.01, P < 0.01, respectively), while the CFPA patients had the most blood loss (P < 0.01, P < 0.01, P < 0.01, respectively).

Sixteen complications developed in 10 patients (11.8%), including pleural effusion, pyothorax, pneumonia, acute respiratory failure, bronchopleural fistula, prolonged air leak, and persistent chest distress. Pleural effusion appeared in 1 SA patient. Pleural effusion and pyothorax were observed in 1 CCPA patient, while another CCPA patient experienced prolonged air leak for 3 weeks. 5 CFPA patients suffered from complications, which makes the highest incidence of postoperative complications, including prolonged air leak, pneumonia (n = 2), pneumonia and pyothorax, acute respiratory failure and pneumonia, bronchopleural fistula. Pneumonia and pleural effusion, and prolonged air leak were observed in 2 AN patients, respectively. No perioperative mortality occurred. Univariable risk factor analyses reveal that CFPA (P = 0.01), longer surgical time (P = 0.001), and more intraoperative blood loss (P = 0.004) were the risk factors of postoperative complication. Logistic analyses show that more intraoperative blood loss (> 400 ml) was the independent risk factor (Table 3).

**Table 3:** The Binary logistic regression analysis for risk factors of complications in CPA patients

Characteristics	Type of CPA	Surgical procedures	Time of surgery	VATS	Intraoperative blood loss
OR	1.37	1.20	3.66	2.21	13.5
95% CI	0.55–3.38	0.72–1.99	0.55–24.53	0.40–12.19	1.63–112.13
P value	0.50	0.50	0.18	0.36	0.02

The majority of patients (n = 66, 77.6%) received antifungal treatment postoperatively, and the length of the medication varied from 2 weeks to 6 months. Voriconazole was the most commonly used antifungal medication.

The median follow-up time after surgery was 47 months. Overall, 6 patients relapsed after surgery with a recurrence rate of 7.1%, all of whom accepted postoperative antifungal treatment without postoperative complications. The mean time to relapse was 14.8 months (2–30 months) after surgery. Relapse occurred in 2 SA patients, 3 CCPA, and 1 CFPA, respectively, while none of the AN patients relapsed. The characteristics of the 6 patients are shown in Additional file 1: Table S1. No risk factor of relapse was found in univariable analyses.

## Discussion

Generally, medical treatment with oral azole drugs is the first choice when CPA is diagnosed. Sometimes the drug course is long and the effect is not ideal. Some authors reported that a considerable proportion of patients do not respond to antifungal therapy, the effective rate ranges from 32 to 76% [7–11]. Relapses occur often after treatment is stopped, in approximately one-third of cases. Many patients may require long-term or even lifelong antifungal therapy [12]. Surgery offers a chance of cure, which has been confirmed by many analyses [13]. However, it is associated with high morbidity and mortality rates due to severe pleural adhesion and infection in the underlying lung. There is general agreement that surgery is limited to patients with a single aspergilloma or with nonprogressive CPA, localized disease, and no significant comorbidities, especially for those with recurrent hemoptysis despite bronchial artery embolization or with azole-resistant disease [3, 14, 15]. Nevertheless, most experience of surgical treatment for CPA is derived from patients with simple pulmonary aspergilloma, with rare studies involving other types of CPA. In this study, we analyzed the surgical treatment of different types of CPA according to the 2015 ESCMID/ERS guidelines [5].

As antifungal treatment is ineffective for SA usually, asymptomatic SA patients should be observed under computed tomography at regular intervals. And surgery should be selected without hesitation when occurring recurrent hemoptysis. Chen reported that 96 SA patients received surgical treatment carrying a complication rate of 8.3% [16]. Complications occurred in 4% of SA patients in our study, the complication rate is lower than others’ [15]. We believe that surgical treatment for SA is safe and effective.

Patients with CCPA without clinical symptoms can also be observed without antifungal therapy and followed every 3–6 months, and should be treated with a minimum of 6 months of antifungal therapy when the disease is developed [17]. Surgery can be considered in CCPA with severe hemoptysis or failed therapy, and studies have reported higher postoperative complication rates in patients with CCPA. In our study, 24 CCPA patients (68.6%) received surgery because of persistent or repeated hemoptysis, 5 of whom experienced life-threaten hemoptysis and accepted bronchial artery embolization before surgery. The postoperative complication rate and recurrence rate were 5.7% and 8.6%, respectively. Some articles have reported that the postoperative outcomes of CCPA are less favorable than those with single aspergilloma, which is not consistent with ours, and we get a similar postoperative complication and recurrence rate [13, 15, 18].

CFPA is the terminal fibrosing evolution of CCPA, and the optimal treatment is not yet known. Continuous antifungal therapy is usually required to delay disease progression or alleviate symptoms. Surgical outcomes are uncertain, and there are only a few reports on the surgical treatment of CFPA [19–21], which may be because of the high postoperative complication rate and mortality. 13 patients accepted surgery in the present study, and 5 (38.5%) suffered postoperative complications, 1 relapsed. We also found CFPA was the risk factor for postoperative complications. CFPA patients usually tend to have more severe thoracic adhesions, more difficult dissections in our experience, and more intraoperative bleeding, meanwhile, we found more intraoperative blood loss (> 400 ml) was the independent risk factor of complication. This may explain why CFPA patients had a higher complication rate.

Aspergillus nodules are usually incidentally discovered on thoracic CT examination, which are very similar in appearance to malignancy and can only be definitively diagnosed through histology [5, 22]. However, only 2 patients were asymptomatic, while 3 had cough and sputum, and 7 suffered hemoptysis. It is not very clear if surgery or other treatment are needed when they are asymptomatic. We performed surgery for 4 patients because of suspicion of lung cancer. Usually, serial follow-up with low dose chest CT scan is suggested for asymptomatic patients in our department.

Open thoracic surgery is often the first choice before, while more and more VATS are performed recently. We performed VATS for 43.6% of patients, and the SA and AN patients were the preferred choices for VATS. The present study confirmed that the VATS surgery was safe and effective and not associated with a higher complication rate and recurrence rate, which is consistent with the previous report [23]. In our opinion, the possible reason is that VATS is often performed for selected patients whose operations are considered to be not very difficult.

Hemoptysis is the most common symptom associated with CPA. In our study, 65.9% of patients suffered from hemoptysis. Life-threatening hemoptysis requires emergency surgery or catheter embolization of bronchial arteries [24, 25]. Bronchial artery embolization is believed to be life-saving for severe hemoptysis, prior to surgery. However, it’s rarely completely effective with a high recurrence rate, it is rather a bridge towards elective surgery [5]. In our present study, 2 received emergency surgery because of massive hemoptysis, while 9 accepted bronchial artery embolization, all of whom relapsed and finally received surgery. This conforms with previous literature [26]. There was no difference among the four groups about hemoptysis and interventional therapy, meanwhile, we didn’t confirm that preoperative bronchial artery embolization would increase or decrease the complication rate and recurrence rate.

The role of antifungal therapy following surgery for CPA cases is controversial [27]. Various studies about antifungal therapy have shown no positive findings regarding antifungal therapy and surgery outcomes [23, 28]. We also didn’t find relationship between antifungal therapy and recurrence in this study. 6 patients who received antifungal therapy relapsed, while none relapsed among the 18 patients who didn’t receive antifungal therapy. However, Setianingrum F analyzed three different time points of antifungal therapy (before, in, and after surgery) correlated with the clinical outcomes of patients, and concluded that antifungal therapy at any point before surgery was highly protective of relapse (P = 0.002), in contrast to a lack of significant benefit if only given after surgery (P = 0.15) [29]. Another study also found the benefit of antifungal therapy given 2 weeks before surgery and 3 months after surgery, but the number of patients was limited [30].

In our view, CPA, as a benign disease, is operated on to eliminate symptoms and preserve more lung function, rather than radical resection as a malignant tumor. So, we resected the main lesion only without secondary lesions in the adjacent lobes for some CFPA and CCPA patients. In the present study, 1 CFPA patient who received lobectomy, 1 CCPA patient who received wedge resection, and 1 SA patient who received wedge resection relapsed. Insufficient surgical resection may be a risk factor for relapse. In addition, high aspergillus spore density may be another risk factor for relapse. However, we cannot quantify these risks precisely because of the lack of relevant data in this retrospective study.

Its retrospective nature and the limited sample size which inevitably introduced bias in the study. We didn’t conclude the risk factor of recurrence perhaps because of its small size. Further large sample studies are required to extend the knowledge on surgical treatment for CPA.

## Conclusion

In conclusion, Surgical resection seems safe and effective in the treatment of SA, AN, CCPA with a low complication and recurrence rate, while surgery for CFPA should be limited to selected patients because of its higher complication rate. We advocate early surgical intervention for SA, AN, CCPA if the patient is a suitable candidate for operation and the lesion could be resected completely, even if the patient has minor hemoptysis. Surgery offers the only chance of cure, whereas non-operative treatment is only considered in the patient who is not suitable for surgical resection. VATS is safe for selected patients.

## Supplementary Information

**Additional file 1: Table S1**. Characteristics of 6 relapsed patients.

## Data Availability

The datasets used and/or analyzed during the current study are available from the corresponding author on reasonable request.

## Abbreviations

CPA: Chronic pulmonary aspergillosis
SA: Single (simple) pulmonary aspergilloma
CCPA: Chronic cavitary pulmonary aspergillosis
CFPA: Chronic fibrosing pulmonary aspergillosis
AN: Aspergillus nodule
SAIA: Subacute invasive aspergillosis
