# Supplementary material for: Outcomes of surgery for different types of chronic pulmonary aspergillosis: results from a single-center, retrospective cohort study
Source: BMC Pulm Med. 2022 Jan 19;22:40. doi: 10.1186/s12890-022-01836-z (PMC8772183; doi:10.1186/s12890-022-01836-z)
Supplement: Supplementary file 1 — Additional file 1: Table S1. Characteristics of 6 relapsed patients. [file 12890_2022_1836_MOESM1_ESM.docx]

Supplemental Table 1 Characteristics of 6 relapsed patients

| Age (years) | Sex | CPA type | Surgical procedure | Postoperative complication | Postoperative antifungal therapy | Time to recurrence |
| --- | --- | --- | --- | --- | --- | --- |
| 48 | Male | CFPA | Lobectomy | No | Yes | 20 months |
| 48 | Male | CCPA | Wedge resection | No | Yes | 2 months |
| 26 | Female | CCPA | Lobectomy | No | Yes | 7 months |
| 44 | Female | SA | Wedge resection | No | Yes | 14 months |
| 51 | Female | SA | Lobectomy | No | Yes | 16 months |
| 54 | Female | CCPA | Lobectomy | No | Yes | 30 months |
